# Thoughts on the Nature and Treatment of Several Severe Diseases of the Human Body

**Published:** 1847-07-01

**Authors:** 


### Thoughts on the Nature and Treatment of several severe Diseases of the Human Body. By *Edward J. Seymour*, M.D., F.R.S., &c. &c. Vol. I. Octavo, pp. 260. London: Longman & Co. 1847.

The contents of this volume consist of four papers on the interesting subjects of—Diseases of the Stomach—Gout—Mental Derangement—and Sciatica. We shall at once proceed to examine the observations of our author upon each in succession, selecting those parts that are of most value, and pointing out, as we go along, what seem to be the chief merits and defects of the work.

The first chapter might have been more appropriately entitled, "Thoughts or observations on some of the more common symptoms of Diseases of the Stomach;" this being the real nature and scope of its contents. The first symptom that is considered is *pain*, and uneasiness in the epigastric region. It is technically known by the names of *cardialgia, gastrodynia, gastralgia,* &c. The cause is very different in different cases. Very often it is dependent upon an undue amount of acid secretion in the stomach. In such cases, there is not unfrequently, at the same time, great irritability of the heart, giving rise to many symptoms simulating those of organic disease of this organ. Inordinate pulsation in the epigastric region is a common accompaniment of this state. All the symptoms, however, give way to the use of antacids with sedatives, or of mild stomachic medicines. Dr. Seymour gives the preference to a powder composed of *rhubarb, calumba, cinnamon,* and carbonate of *soda*—taken before dinner or at bedtime, for a fortnight at least.

When the cardialgia is not accompanied with heartburn or vomiting, "it may be believed to arise from the stomach being thrown into irregular contraction." The best remedy in such a case is a combination of the trisnitrate of *bismuth* and *magnesia.* The *hydrocyanic acid,* also, may often be used with much advantage. If the bowels are torpid, mild aperients will be required every second night or so.

> "Where the pain has resisted this treatment, and the emaciation is very great, so as to give alarm lest there should exist organic disease, as of fungus or cancer, the best remedy is a grain of *opium* thrice daily, the bowels being kept open by means of injections; the food being animal principally, and in *small quantity.*" P. 7.

The case of the poor Italian, quoted in illustration of this practice, is set down as an example "of over-secretion of acid in the stomach," cured by *opium.* It might be so; but the report of the case does not bear this opinion out.

Pain of the stomach immediately after taking food is not unfrequently indicative of gouty derangement of the system. Even then, Dr. Seymour appears to think highly of the use of opium (a grain three times a day), the bowels being kept open by enemata. From its well-known effects of usually increasing the formation of urea and uric acid, it will be always right to associate its use with that of alkaline medicines.

*Pyrosis* or waterbrash, when dependent upon a merely functional derangement of the stomach, is best treated with the compound *kino* powder, gr. v. ter die; the diet to be light and nourishing, and weak brandy and water allowed as a beverage. This symptom, however, is not unfrequently an accompaniment of medullary carcinoma of the stomach—a form of cancer, which, alas! often attains to a most serious extent, before the presence of organic mischief has been even so much as suspected. "It is, therefore," Dr. Seymour observes, "incumbent on the physician, when there is waterbrash, and especially if the patient be upwards of thirty, to enquire particularly as to the state of the epigastrium."

Another cause of severe pain at the pit of the stomach is the presence of a gall-stone, either in the gall-bladder, or in its passage along one of the bile-ducts. As a matter of course, if the free excretion of the bile is interrupted, symptoms of jaundice supervene; but, as long as there is no decided obstruction, the fæces and urine may retain their healthy colour, and the skin not exhibit any icteric tinge.

In another set of cases, which generally occur in those—women more especially—who are "broken by servitude or distressed by wayward affections," the pain may be indicative of Ulceration of the Stomach going on to perforation. This lesion often gives rise to very profuse hæmatemesis. The following is the description which our author gives of the usual course of such a case :

" The chronic ulcer of the stomach is not to be distinguished by any known signs, unless vomiting of blood, which has followed long-continued symptoms of pain and distress in the stomach, has taken place. Up to this time, it is easier to say what the disease of the stomach is not, than what it is.

" The patient has long felt pain after eating, without the symptoms of the presence of acid which I have before described. At one time one species of aliment, at other times another, seems to aggravate the symptoms. Solid meat is generally felt to be most distressing; and the state of emptiness is the state accompanied with the least pain, as in a case related by Mr. Travers in the Transactions of the Medical and Chirurgical Society. Suddenly the symptom of pain becomes accompanied by constant vomiting and a painful burning of the epigastrium, with extreme pain in the opposite vertebræ; the pulse becomes very small and weak; and cold extremities and shrinking features denote the approach of death, which occurs in a few hours after the aggravation of the symptoms: and on examining the body, perforation of the stomach,—simple nearly circular ulceration, without any thickening of the edges, without any inflammation of the surrounding mucous membrane,—is seen. In such cases the patient has not lost flesh, as in the case of cancerous or fungoid disease. No examination, however careful, can distinguish before death either hardness or fulness; nor has the appearance of the tongue shewn by its bright redness that serious irritation is established in the stomach; neither does the external surface of the body present the straw-coloured appearance, arising not only from the absence of red blood, but as if the vessels were filled with size." P. 18.

Life is sometimes prolonged for weeks, and even months, after perforation of the stomach has actually taken place; adhesions having already been formed, so as to prevent the ready extravasation of its contents into the abdominal cavity. Dr. S. relates two cases: in one, the patient survived twelve days, and in the other five months. Both were young females.

When, from the preceding pain and subsequent vomiting of blood there is reason to suspect the presence of simple ulceration of the stomach, Dr. Seymour strongly recommends the internal use of the oil of *turpentine* —ʒij., with as much castor oil, being taken in the form of an emulsion, every, or every second, morning.

" I have never," says he, " seen a case which did not recover for the time being from this practice. In three cases where the patients had suffered from this disease, and the hæmorrhage had entirely ceased under the use of the oil of turpentine, healed ulcers, into which small branches of arteries could be traced, were observed on the surface, I believe, in all, on the posterior surface of the stomach; but in a great many more cases, where the patients are still living after a lapse of several years, and where of course the case cannot be proved, the disease gave way to the use of the oil of turpentine. Not only were large quantities of blood vomited in such cases, but large quantities passed half digested from the bowels, under the form of thick matter, resembling soot in appearance. I do not remember to have witnessed a single case in which this treatment has failed; and, notwithstanding that the medicine is very nauseous, it rarely happens that it is rejected by vomiting.

" I am not, however, to be understood to say that hæmatemesis always arises from ulceration of the stomach; that it does so in some instances has been proved; in others it may arise from simple exudation, or from vicarious menstruation; occasionally from diseased liver; and occasionally from fungoid or cancerous disease of the stomach." P. 27.

If the turpentine excite much nausea, or otherwise disagree, the diluted *sulphuric acid* or the *acetate of lead* may be given; but neither of these medicines is equal in efficacy to the turpentine. The *liquor stypticus* of Ruspini has been used with decided advantage—ʒij. every four or six hours.

At this part of his subject, our author flies off to the consideration of that most hackneyed subject—"Diet in general." There is nothing, however, in his remarks that deserve notice, although he has favoured his young friends in the profession with a recipe for making beef-tea, which he purchased from a first-rate French cook! We pass on, therefore, to the notice of another common symptom in stomach-complaints, viz. *Vomiting.* Every medical man knows that this is a frequent, and sometimes not only a distressing but even an alarming, accompaniment of early Pregnancy. It is then often best relieved by one or two small blood-lettings. At other times, the use of *opium,* alone or in an effervescing draught, will be found most effectual. A mustard poultice, or a blister, to the epigastrium will often succeed. Confining the patient to a diet of equal parts of milk and lime-water has answered, after other remedies had failed. The vomiting of Hysteria has sometimes yielded most speedily to *creosote* ♏j.—ij. ter die. Minute doses of *strychnine* have been much recommended by some writers in obstinate hysterical and parturient vomiting.

Dr. Seymour makes the remark that "vomiting in Phthisis, occurring almost always after coughing, if it arise early in the disease, is the proof of a severe and rapid form of it; if late, it betokens that large collections of matter are locked up in the lungs, that is, have not yet found an outlet through the larger branches of the bronchi."

He briefly relates one case, and refers to several others, in which an accurate diagnosis was formed from attention to the symptom now mentioned. Dr. S. might surely have illustrated this point—one, according to him, of much practical importance—with greater minuteness and circumstantiality of detail. Equally unsatisfactory are his remarks on the Iliac Passion, of which he has seen three cases, in all of which recovery took place. Strange to say, he does not as much as allude to the use of enemata for the purpose of inducing "a strong (peristaltic) impression downwards,"* in the obstinate vomiting, which is the worst symptom in this distressing disease, as well as in Carcinoma of the stomach; although he thinks it worth while to tell us that the late Dr. Cholmely was in the habit of using crude mercury (in doses of ℥iv) for the purpose, and that "in a very obstinate case of vomiting, also from organic disease, great relief was obtained *temporarily* from a scruple of calomel prescribed by Sir. H. Halford."

---

* We have repeatedly found the most obstinate vomiting give way to the use of a purgative injection, leaving the stomach entirely quiet, without either food or medicine being given for six, twelve, or twenty-four hours. The application, at the same time, of a mustard poultice to the epigastric region will often very materially assist in checking the inverted peristaltic action.

Tubercular Disease of the Peritoneum is generally attended with very troublesome vomiting; and the appearance of the vomited matter is in many cases so peculiar, that it affords, in our author's opinion, a diagnostic sign of the disease. It is of "a green so dark that it is only to be compared with that which the sea acquires at great depths,—a blue as intense nearly as that of indigo; deep green when regarded in one way, blue in the other." The disease may exist without this symptom; but, "when this symptom is present, it is diagnostic of the disease." We must not omit to mention that Dr. Seymour maintains that tubercular disease of the peritoneum is of much more frequent occurrence than Mesenteric Disease. Nay, he goes so far as to say, that "mesenteric disease is very rare, and the disease in question by no means so; in fact, in hospital and private practice, where I have had occasion to verify the disease after death, this occurs at the least, in proportion to mesenteric disease, as five to one; and, I believe, in a still higher ratio."

The experience of most physicians will certainly not agree with that of our author as to the great rarity of mesenteric disease in children.

Vomiting, often of the most intractable description, is a conspicuous symptom of the passage of a Calculus along the Ureter. It is, of course, very important to distinguish the true cause, from the first.

"The distinction is to be found in the fixed pain in the direction of the ureter, most frequently about midway between the umbilicus and the spine of the ilium, but still more in the slow soft pulse, notwithstanding the intense pain which accompanies the disease. In inflammation, the universal pain over the abdomen, the patient carefully keeping the trunk of the body at rest, the pulse being small, hard, and very quiet, the alteration of the features, well distinguish it from the acute fixed pain, the constant vomiting, the throwing about of the limbs, the numbness in the thigh of one side, the patient seeking the half-recumbent posture, which appears to relieve the pain, which constitute the form and the picture of renal colic. The sickness, however, in females especially, is often frightful. I have seen it, almost without ceasing, continue for eight days consecutively, but there was no fever, no constipation, no wasting of the body; and a loaded state of the urine, or the passage of a small calculus, soon put a stop to the symptoms." P. 55.

Our author next alludes to the vomiting which attends Cholera, the Asiatic as well as the common form. He briefly says, with respect to the latter, that, "almost always, the mixture of three grains of calomel with a grain of opium, will arrest the disease;" and straightway he tells us a story about a young gentleman curing his friend at Padua, by this prescription! So infallible are its virtues in the estimation of our author, that he thinks it worth while to put upon record this interesting piece of information:—

"For many years I have been in the habit of giving this prescription to friends of mine, or members of my family, when on foreign tours, and very often they have had occasion for its use: never has any evil resulted, nor has it failed of its efficacy in a single instance. To my knowledge, in the course of the last twenty years, the number who have benefitted by it is very great." P. 59.

After this, who will be surprised to hear of "Dr. Seymour's Pills" for the use of travellers?

The last ten pages of this paper are occupied with rambling remarks on *gastrite*, and softening of the mucous membrane of the stomach; but there is nothing in them of the slightest value.

The next subject that Dr. Seymour discusses is Gout. No fewer than 80 pages are devoted to its consideration. Dr. S. acknowledges that there are "hundreds of books" on the subject; but still he thinks that something remains undone.

"I do not know," says he, "that I can say anything new, but the arrangement of our present knowledge on the subject, our treatment and the reasons for that treatment, are not satisfactorily stated, in my own view of the case, concisely and for practical use. It is to supply this (temporary if it be) want, that having experience on this subject, I undertake the task of making the following observations." P. 72.

Let us now see what is the nature of these observations. After a brief historical sketch of the disease as known to the Greeks and Arabians, the question "what is gout" is propounded for solution. The old physicians, it is well known, "recognised a specific morbid fluid mixed with the blood, which was thrown off upon the joints, thus purifying the blood, and producing in its elimination what we now call an inflammatory condition of the joints and tendons." Dr. Seymour's account of the modern theories of the disease is certainly not very lucid. He thus describes the opinions of English and French physicians in the present day.

"1st. The opinions popularly held in this country attribute the gout to vicious secretions of the stomach and liver; after some time, these derangements are followed by inflammation in the feet or hands, such inflammation being a natural termination or consequence of this disorder of the viscera, and of their suppressed or vitiated secretions. This is most generally the only explanation to be derived from those who have been taught in early life, that all diseases of the human body depend on disease or disorder of the secretions of the liver or of the bowels.

"2nd. The opinion most prevalent in France is, that the cause of gout is the indulgence in too succulent and nutritious a diet.* All the textures of the body become gorged, and more nourishment is afforded them than can be removed by excess of excretion. Two modes of excretion, the urinary discharge and the cutaneous perspiration, keep up for some time the equilibrium between the ingesta and egesta, but sooner or later it happens that these outlets are not sufficient, or one of them is completely obstructed by some temporary cause, and thus the over-nutritious particles, which ought to be carried out of the body, are transported to the fibrous articular structures. The nutrition is greatly increased in these structures, otherwise almost insensible, endowing them in some measure with a new species of vitality. They are transformed from parts formed for motion and passive resistance to sensible and irritable parts, no longer able to fulfil these functions, and disposed to become inflamed spontaneously, or from an ordinary exciting cause. Again, these nutritious materials in excess are deposited on the surface of the articular structures, and form the concretions composed in the greatest part of animal substance, as they for the most part consist of azote and animal matter.

"3rd. A still more modern theory supposes that the acid matter which is

---

* "The over animalization of the blood, consequent on an over nutritious diet, redundance of thick and viscid bile, explains the frequency of biliary calculi and gout. The secretion of bile affords one exit for the overnourished fluids of the body; still more the urine, and the highly loaded urine in gouty persons, sufficiently attests the state of the blood, which is far more than able to supply the necessary secretions in their normal state, and the nutrition of the body."

distinguished in the urine and in the perspiration, and in the concretions in the joints, is formed in the blood, and very recently the changes in the blood produced by electricity are believed to form, in the living body, this acid so uniformly present in gouty excretions." P. 77.

We really were not aware that British physicians generally attributed the disease merely to " vicious secretions of the stomach and liver," nor the French to mere plethora—not *congestion*, as Dr. S. inaccurately terms it. This is an off-hand way of writing that is surely very objectionable; nor can we admire the accuracy of an author, who tells us that, gouty concretions " consist, for the most part, of azote and animal matter." Dr. Seymour's explanation of the hereditariness of gouty complaints is given in these words :

" The blood is more highly animalised, and filled with noxious particles, which are thrown off by inflammation of the extremities, termed gout, when no longer to be borne; and this state and condition, when children are procreated, may be transmitted to them, as scrofulous virus and syphilitic virus notoriously are." P. 78.

But is there not this very marked difference ?—that, whereas the signs of what our author calls the scrofulous *virus*, as well as of syphilis, are manifest in a child from its birth, those of gout seldom shew themselves before the period of adolescence ? Dr. Seymour is scarcely correct even with respect to the ordinary exciting cause or causes of the disease in question. Is it really the case that those, whose blood is most highly animalised by " eating succulent food, with habits of a sedentary kind," are most subject to it? Such persons may become excessively full, bloated, and plethoric ; but, if they have not indulged in the use of fermented or vinous liquors, they will very rarely indeed become gouty. In short, it is less what is eaten than what is drunk that predisposes to the disease. *Spirit* " drunkards, simply speaking, are not gouty :" Every one knows that. But can the same thing be said of those who are, or have been, in the habit of drinking their strong ales, and stronger wines, or both of them, as is often the case, together. No wonder that the only gouty patients to be seen in hospitals, are " decayed butlers and housekeepers." Dr. Seymour tells us that he once admitted four cases of gout into St. George's Hospital on the same day; and he goes on to say that he bid one of his pupils " ask those patients, quietly, how much meat they have been in the habit of eating daily ?" The answer was, in one case, three times; in all the rest, twice daily, and largely. ' And how often had they left the house ?' ' Not for weeks together !' "

True ; but had not these butlers and housekeepers, besides their fat feeding and their lazy habits, been tippling daily with their wine and ale ? The last element is quite as necessary as the two former for the production of the malady. It would be unprofitable to follow Dr. Seymour in his account of the mode of attack, and of the symptoms of gout, as there is nothing in it but what is perfectly well known to every reader. One observation only calls for notice. It is this :

" Where patients die, worn out as it is called with gout before arriving at a great age, I have generally seen enlargement of the heart (hypertrophy), and this was signified in the latter months of life by anasarca." P. 87.

The Hypertrophy in such cases is almost invariably associated with Dilatation of the heart at the same time. Anasarca can scarcely be called an indication of simple hypertrophy.

With respect to the treatment of Gout, Dr. Seymour has thought it worth while to enter into a minute account of a variety of the remedies that are, and have been, most in vogue. Many pages are devoted to the history of the *Eau medicinale*, and of *colchicum* and its preparations. As to the probable modus operandi of the latter, all that we are told is to the effect that " Sir Everard Home wrote a paper in the Philosophical Transactions for 1816, to prove that the efficacy of colchicum, resulted from its being communicated through the blood." What may be the exact meaning of this remark, it is not quite easy to determine. At all events, it might surely have been expected that some reference would be made to the not unimportant fact of the quantity of the urea, and probably also of the uric acid, being found to be decidedly increased during the exhibition of the remedy. Again, was it worth while to enter into such particulars about the " Portland powder," the " Chelsea Pensioner gout powder," &c., &c. ? Formulas for both are given. And here we cannot but remark that most professional readers must be offended by the gossiping tone, that pervades much of our author's writing. For example, we have scraps from Horace Walpole's letters about his bootikins, Sir W. Temple's account of the *moxa*, anecdotes about the use of *burdock* tea, and so forth; and, when mention is made of the effects of the Weisbaden and Carlsbad waters in the convalescent stage of the disease, we are told that

> " Their great heat, combined with the large quantity of alkali contained in them, makes them well adapted to such cases; and twenty-five years ago, in Italy, I frequently met with men of rank from the North of Europe suffering from calculous diseases in a greater or less degree, who made it a rule to visit Carlsbad annually for the sake of their health, and passed during their stay small calculi of lithic acid, while correcting the disposition to this secretion: and I well remember a Russian Nobleman, of rank so high as to have been Ambassador-Extraordinary to this court, who assured me he had gone several successive years to Carlsbad, and shewed me a large number of calculi of lithic acid of the size of small melon seeds, which he had passed each year while drinking the waters." P. 117.

The subject of Hydropathy comes in for notice, and is ushered in with the announcement that " M. Priessnitz has become the head of a new sect of medical philosophers (!)," and a recommendation of *Dr.* Herbert Mayo's book upon the subject. This gentleman, " who has witnessed this practice, says, as far as regards our countrymen, that the taking large quantities fasting (say a pint or two) has disagreed with many. He thinks, however, that by substituting large quantities of water during the day, and especially at meals, in the place of wine, or stimuli, not only is the health greatly improved, but that the gout may be *cured*. He expresses his conviction of this fact. But the system has been established too short a time for certainty. What is the period of ten years or fifteen years in the life of man sufficient to be *secure* from an attack of gout, or to establish a certainty ? and yet this period of time has scarcely elapsed since the promulgation of the new treatment."

The chapter closes with some remarks on the forms of Cutaneous Disease which are frequently met with in gouty habits. Psoriasis is that which is most commonly met with. In many cases it is associated, especially in the extremities, with an oozing of pure firm fluid from the affected surface; so that the eruption may be truly said to be an impetiginous psoriasis; constituting, as all know, a very distressing and unmanageable complaint.

"Local applications are injurious; the mildest only can be endured. The sprinkling the part with hair powder, and the application of the Unguent. Acet. Plumbi, can best be borne.* But, internally, the remedy from which I have seen the most satisfactory effect is from the use of *pitch* in large doses.

"I will not diverge here to remind the reader of the great efficacy attached to tar water as a stomachic in the last century, nor recal to his mind that *some advantage* may be derived from a remedy so long used and so much praised: but I can assert from much personal experience that the use of this remedy is most effectual—that, both in the hospital and out of it, I have seen cases of the most decided cutaneous mischief cured by the use of tar. It is more effectual when the disease appears to be connected with disorders of the stomach, and the more as it approaches in appearance externally to the scaly or squamous eruption. Unfortunately for its popularity, it must be taken for a long time together, and in large quantities: on the other hand, this is the only inconvenience which results from its employment.

"In no instance has it ever produced, in my experience, when taken for several months, the smallest bad effects. On the contrary, the general health improves: if any obvious effect be produced, excepting the cure, it occasions a slightly relaxed state of the bowels, or some perspiration.

"The following is the formula which I use:

℞ Picis Liquidæ ℈ij.

Pulv. Glycirrhizæ ℈j. M.

Ft. pilulæ xvi. æquales quarum capiat duas vel tres ter in die." P. 145.

In some cases, the *arsenical* solution internally, has been used with much advantage; chiefly when the eruption is squamous rather than pustular. The decoction of the *dulcamara* has been generally considered a good vehicle for it.

The Vesicular or rather Bullar form of eruption, known by the name of Pompholyx, is occasionally met with in gouty patients, and sometimes proves very troublesome.

"The body, but especially the arms and legs, are covered with vesicles or bullæ from the size of a pin's head to that of a large pea, and even of a larger size. There is no fever, these vesicles break and discharge, leaving deep scales. The patient thinks he is getting better, when all at once, in a single night, another crop of bullæ make their appearance. In two cases, after such an attack, the patient was free from gout, in one instance five, in the other six years.

"Arsenic alone has no effect on this disease, nor has mercury; external applications, from the extent of the surface, are manifestly inapplicable. But what these severe alterative remedies will not do alone, they effect when taken during the same course. Thus, I have seen the most obstinate cases speedily give way under five minims of Liquor Arsenicalis, twice in the day, in distilled water, and from three to five grains of the blue-pill at bed-time, with or without a pur-

* The decoction of poppies, to which a small portion of Goulard extract is added, and applied tepid, will often be found more soothing than any other application. The addition of the Hydrocyanic acid also is, in many cases, very useful.

gative as the bowels may require: or the blue-pill may be given on alternate nights.

"I have seen cases which had been very troublesome for several months, cured in little more than a week by this practice, although the remedies separately had been already tried without effect." P. 147.

In the treatment of this, as well as of every other, form of cutaneous disease, more especially if occurring in a gouty habit of body, the physician will do well to have his attention directed to the state of the urine. When this is in a healthy condition, recourse may be had to the use of the *hydriodate of potash* with excellent effects in a great variety of skin complaints. It is most advantageously given with *sarsaparilla*.

From Gout we shall pass on to Sciatica, the subject of the 4th Chapter, leaving that on Mental Derangement to be last considered. This is altogether better, seeing that, in not a few cases, there is some alliance between these two kinds of bodily suffering. The definition of Sciatica by our author—a pain greater or less affecting the great sciatic nerve, or *even when affecting large nerves of the body*—is certainly quite comprehensive enough. He describes three forms, or rather degrees, of the malady.

"In many cases the patient only complains of a dull heavy pain at the back part of the thigh, most severe in damp weather, severe after walking, and especially on ascending a stair, often increased after a full meal, and principally after the limb has been cramped by sitting. This is the milder form of disease.

"In the second form the low grumbling pain in the course of the nerve is scarcely ever absent, but in addition violent accessions come on, with comparatively long intervals of ease. In the paroxysm the pain is described as if a pistol-shot discharged down the course of the nerve, and the suffering is extreme. This is the second degree of the disease.

"Lastly, the patient is quite unable to move the limb; when moved, the pain becomes excruciating to so great a degree, that the patient will not willingly permit a friend to approach his bed, or walk quickly on the floor, lest the movement of surrounding objects, or of the bed, should renew the pain.

"Nor is this all: generally at night, most frequently about midnight, violent pains come on in the nerve; the limb, formerly motionless, now shakes so that even force can hardly restrain it; the muscles and tendons are affected by the cramp, and the unhappy patient lies screaming often for several hours in torture. Towards morning the pain becomes diminished, and the patient falls exhausted into slumbers, without which it appears to be difficult to conceive how life could be prolonged; for such paroxysms sometimes recur nightly for weeks." P. 226.

As a matter of course, the first and most important part of the physician's duty is to try to form an accurate diagnosis of the true nature and cause of the malady he seeks to relieve. He must therefore seek to discover whether it is simply and purely a local affection of the sciatic nerve, or whether the pain be but sympathetic of disease elsewhere. There are two diseases for which it is very apt to be mistaken; 1, disease of the hip-joint; and 2, diseased or disordered condition of the kidney, especially that state in which there is an excess of uric acid in the urine. Whenever sciatica occurs in a person who has suffered from the gout, we have reason to suspect that it is connected with this truly systemic disorder; and it should be treated accordingly. "The various preparations of *colchicum* will be of great importance; but in the cases which I have witnessed (and they were very severe), *quinæ sulphas* in large doses given in the day time, with thirty drops of the wine of colchicum in a draught at night, were most effectual; but neither separately produced permanent relief."

Some very obstinate cases of Sciatica appear to be connected with a syphilitic taint; hence the continued use of *sarsaparilla, iodine,* &c. has proved successful, when all other remedies have failed.

When the disease is ascertained to be quite unconnected with any constitutional depravity, such as gout and syphilis, or with any disorder of remote organs, such as of the stomach, kidneys, &c. and when, therefore, there is just reason to believe that it is purely local, the use of topical remedies will form an important part of the treatment. Cotunnius was of opinion that the principal cause of the malady was an effusion of fluid into the sheath of the nerve; and accordingly his favourite remedies were leeches, cupping, and blistering, with the view of causing the absorption of this supposed fluid. Dr. Seymour appears to have found more decided advantage from the use of *acupuncture* than from any other remedy in severe sciatica, not referable to constitutional disorders. Its modus operandi is hinted at in these words: "If the opinion of Cotunnius as to the pathology be correct, the benefit arises from setting free the fluid contained in the sheath of the nerve. I am inclined to be of this opinion." The remedy truly is better than the interpretation thereof.

Of the class of sedatives, *belladonna, opium,* and *aconite* have, on the whole, been found most useful. The first, in the form of extract, has been used in many cases with decided advantage, especially when the disease appears in young females, and is connected with an hysterical state of the constitution. The dose is from one-sixth to one-third of a grain; its effects upon the system must be watched. "It undoubtedly," says Dr. S., "exercises great influence over nervous pain. I have seen it control local spasms of the face especially, and spasmodic loss of voice, in a very remarkable manner; it produces usually, at the same time, symptoms of great depression of vital power. I have seen, more than once, the terrible disease 'tic douloureux' suspended for ten years by its employment." For external use, the extract should be mixed with equal parts, or thereabouts, of soap plaister or mercurial ointment, and applied along the seat of the pain. It should not be used in the endermic method, in consequence of the poisonous effects it might produce by being absorbed.

Dr. Seymour has no great confidence in *aconite*; its effects are unequal, and cannot be depended upon. He has reason to believe that it is apt, like *digitalis* and some other medicines, to accumulate in the system, and give rise to alarming nervous symptoms. The case, however, advanced by him in proof of this is not satisfactory; seeing that the cerebral affection might have resulted from the stoppage of the chronic diarrhœa, for which it had been prescribed.

The use of the alkaloid, *aconitine,* has been highly praised by some practitioners of late years. "It has been said," says our author, "that half-a-drachm of this medicine (gr. xv. Adipis Ppæ. ℥j. Ol Olivæ ʒss.) rubbed through (along ?) the course of this nerve, has been found productive of immediate relief,—a great happiness in extreme cases."

Dr. Pereira recommends for external application a tincture of aconite, prepared by macerating a pound of the root in a gallon and a half of rectified spirit.

It is scarcely necessary to say that, in all cases, when the pain is very severe, returning in paroxysms lasting from one to several hours of almost intolerable suffering, nothing can take the place of *opium* in some form or another. The acetate or muriate of Morphine is perhaps the best form in which it can be administered. The dose required to afford relief is sometimes enormous. Dr. S. mentions a case where as much as six grains of the acetate were obliged to be given during the attack, before any advantage was obtained.

All that we hear respecting *iodine* and it preparations in the treatment of Sciatica is to this effect:—"The well-known effects of this medicine in secondary symptoms would induce its employment where the sciatica appears to arise from a syphilitic taint, but in cases where this painful affection arises as symptomatic of an over-excited state of the nervous system I feel certain it is injurious. In one case, undoubtedly, the employment of it was followed by great increase in the severity of the pain."

It is more especially in Rheumatic and Syphilitic cases that the hydriodate of potash has been used with the most advantage. We have also seen it produce excellent results in neuralgic affections occurring in cachectic states of the system, indicated by the outbreak of impetiginous and ecthymatous eruptions on the skin. It should then be combined with sarsaparilla or colchicum, or with both.

When Sciatica is associated with those marked states of the stomach and bowels, which are accompanied with much flatulence, there is no better remedy than the oil of turpentine. A drachm of it may be given in the form of an emulsion, prepared with the yolk of an egg, and some aromatic water, for several mornings in succession. It may also be administered as an enema in many cases with marked relief, both to the flatulence and the pain.

In his concluding observations, Dr. Seymour alludes to severe sciatica (sometimes one of the brachial nerves is the seat of the pain) being a premonitory, or at least a precursory, sign of Cerebral Disease. There is one symptom which he has reason to believe is usually present in such cases, and the existence of which, therefore, it may be of importance to attend to, as aiding the diagnosis: this is "vomiting after taking any solid food, either provoked voluntarily, or occurring with very little effort." As a general remark, whenever spontaneous vomiting has existed for a length of time, resisting all the usual methods of relief, and when there is no reason to believe any organic mischief of the stomach or of the chylopoietic viscera, the physician should suspect the presence of diseased action in the encephalon. In cases of this sort, the establishment of an issue in the nape of the neck, and the cautious administration of sedatives, regularly and perseveringly employed, may be the means of delaying the progress of the cerebral mischief.

CHAP. IV. *On Mental Derangement, especially that form called Melancholia, or the alternation of melancholia with violence, termed Lypomania or Suicidal Madness, especially in reference to the treatment of this form of disease by the employment of Opiates, or of that class of medicines called Sedatives.*

The following sentence from Esquirol is affixed to this chapter as a motto or epigraph :—"Every organic lesion, that has ever been found in the bodies of the insane, has been observed in those of persons who had never been affected with chronic delirium : in many cases no abnormal change has been discoverable, although the insanity had existed for a number of years. Pathological Anatomy has shewn to us every part of the encephalon altered, suppurated, destroyed, without any chronic injury of the understanding."

The great object of Dr. Seymour in this chapter is to recommend and enforce the great utility of opiates and other sedative and hypnotic medicines in certain forms of mental alienation, where there is reason to believe that no organic lesion of the encephalon is present. The existence of such a lesion may be suspected whenever there is loss of power in any of the limbs, liability to epileptic fits, cephalalgia accompanied by dimness of sight and loss of memory, &c. In all cases of this sort, the prognosis must be most unfavourable.

Passing over the introductory remarks,* which are, it must be confessed, most loosely and carelessly written, we proceed at once to the consideration of the very important practical point that our author seeks to illustrate. His attention was first drawn to the benefit of the use of opiates, continued for a length of time, in certain cases of insanity, about eighteen years ago, by Messrs. Berverly and Phillips the medical superintendents of Dr. Warburton's very extensive lunatic establishment at Bethnal Green. Their statement was to this effect :

"We have found the acetate of morphia useful both in the excited and the low form of insanity. We have also found it useful in cases of fixed delusions, but not of any great standing, and more useful in the low than the excited form of the disease. Of five cases of melancholy, three got well; the remaining two are certainly improving under the use of this medicine. Of five cases of excitement, two were discharged cured; one remains much improved; two received no benefit. It is necessary to observe, that we have used this medicine in several cases without taking notes, and the result was similar to the two cases mentioned, that is, without benefit. It appeared to us, that morphia did not produce the same good effect in excited as in other cases, unless there was an occasional interval of reason. In the cases mentioned, we have commenced with a fourth, and have not found it necessary to exceed half a grain; at present we have a patient taking half a grain dose every night, with decided advantage, and we

---

* Many of the observations at the close too of this paper are scarcely intelligible, from sheer carelessness in the manner of their expression. For example :—"The state of the memory, the principal function of the brain, which depends on the perfection of the organ, is to be carefully considered. In old people the deficiency appears traceable to ossification of the arteries, and the first failure often precedes it." Are we to understand that the decay of the memory, "the principal function of the brain," is owing to incipient ossification of the cerebral arteries ? Where is the proof of this assertion ?

think the case very interesting, and proving the extraordinary effect of the medicine in cases of melancholy.

"A woman, of the age of thirty-six, the mother of four children, was attacked with depression of spirits while pregnant with her last child. She did not feel the attack before she quickened, but immediately after: she had a strong desire to destroy herself and children. This continued during pregnancy. After she was delivered she became worse, and attempted to commit suicide several times; and described her feelings; which is not common in such cases. She continued in this state, not fit to be trusted without a strict watch. She was sent here about two years ago; and what is extraordinary in her case is, that about noon all the feelings of the desire of self-destruction left her. This occurred within the last three months; from which time they have remained the whole of the day. Various means were tried, without effect. Our first idea, from the regularity of the attack, was to treat her disease as an intermittent; which failed. About a fortnight ago we gave her the morphia, beginning with the fourth of a grain, and gradually increasing it to half a grain. After taking the second dose, one-third of a grain, she slept all night; in the morning she was cheerful, without feeling the propensity to destroy herself. The third day she had a return, which lasted until noon; the dose was then increased to half a grain. The fourth morning she had not any return, and continued well until the fifth day after the half-grain dose was given, when she had a return from five o'clock in the morning until nine; a paroxysm three hours shorter than any of the preceding. She is now free from any desire to destroy herself." P. 155.

Since then, Dr. Seymour has employed this practice in very numerous cases, and with most pleasing effects. "Upwards of 70 cases (of Melancholia and Hypochondriasis) have recovered; and I consider no case to be called a recovery, unless two years at least of unabated health have elapsed since the treatment concluded. In nearly 20 cases, the treatment has failed, or only given temporary relief." The preparation employed was the *acetate* of *morphia;* the dose being, in mild cases, a quarter of a grain in the form of solution, and, in severe ones, half a grain increased speedily to one grain, every night at bed-time. The practice must be steadily continued, without the intermission of a single night, *for several weeks in mild cases, and for three months at least in very severe ones.* Sometimes indeed, at first, sleep is not induced; but, almost always, rest and quietude are at once obtained. Slight nausea and disturbance of the head may be felt for the first few mornings; after a short time, however, these unpleasant symptoms cease, the patient sleeps at night, and is free from pain and uneasiness during the day. In some cases, two, three, or four weeks will pass before any marked or decided benefit to the disturbed state of the mind or feelings is experienced. Still, the dose should not be increased beyond what has been already stated. Dr. S. assures us that, even when the treatment has not succeeded in curing the disease, he has never witnessed any ill effects from the use of the Morphia, provided it has been given in the manner which he directs; viz. not to increase the dose beyond that recommended, and to keep the bowels daily open. Occasionally, the application of cold (ice in a bladder) to the head has been employed at the same time; this remedy is chiefly useful when the melancholy and mental depression alternate with paroxysms of violence. The regular employment of the *tepid bath,* every or every second day, is a most serviceable adjuvant in very many cases; particularly in females, and more especially when the disease has arisen in the puerperal state. "The only remedy," says our author, " which could, some thirty years ago, be recommended as being of certain advantage at the Quaker's Retreat at York was the warm bath in melancholia; and the same was stated to me at Charenton in the year 1819.*

There is no form of Insanity more amenable to the treatment, that has now been recommended, than that which arises immediately after Parturition. We give the report of one case:

" About nine years ago I was sent for to Islington to see a lady, who had been confined the day before. This lady had had, a short time before her expected time, a fright from an alarm of fire, and her labour, although some days after the fright, was believed to be on the whole premature: the child was living. The patient's state was dreadful; her screams and cries were under the apprehension that she was condemned, and in hell fire. The lochia had ceased to flow; the patient, young and always delicate, seemed to have almost superhuman force; but her expression and violence of fear were scarcely to be endured. The pulse was 130, or even quicker. I recommended a grain of the acetate of morphia in solution, and a warm bath, every evening; her bowels to be kept open. I did not visit her again for two or three days, and I well remember the astonishment with which the anxious friends assured me she was so much better.

" The practice was continued, and I only saw her once afterwards; the further attendance of a physician being quite unnecessary." P. 174.

When the Insanity has arisen during the early months of Pregnancy, it is usually much more troublesome and unmanageable. " Its character is generally that of self-accusation, alarm or horror for the husband or children; scenes of future punishment often present themselves, and a peculiar dread of taking food for fear of adding to the evil by increasing the health, and thus perpetuating the wickedness of which the patient considers herself to be guilty, is constantly present." There is often a very strong disposition in this unhappy state to commit suicide; and it may require the greatest and most watchful care to prevent this dreadful occurrence. It is not unfrequently supposed that the mental derangement will cease after the labour; but this, in Dr. Seymour's experience, has not been the case; nay rather, " the birth of the child has been the signal for an increase of the disorder, and unless cured—and it may be cured by the means I mention—no alleviation has occurred until after another child has been born." Is this last condition, pray, necessary for the recovery of the patient?

There is much truth in Dr. Seymour's remarks as to the utter inutility —in many cases the positive mischief—of travelling and frequent change of scene upon patients afflicted with this kind of insanity:

" In every case that this course has been pursued, it has been invariably hurtful in my experience. Nothing is so common as to say—' Poor thing! give her or him a little variety; rouse them, change the scene.' In simple hysterical cases this may do, but in cases of real aberration of intellect in melancholy, two circumstances render it wrong.

" First, presenting to a mind impaired a succession of objects too quickly.

" Secondly, that the mind pre-occupied considers all this as an abomination: ' What do they drag me here for? I who am so wretched: ah! it is all very well: what a mockery in my state!'

---

* " *Se melancholiam incipientem solo aquæ dulcis balneo frequenter curasse.*"— Galen de Locis Affect. c. 7.

"Thus it is, and thus it has been forced on my mind, in a great number of examples, both of men and women, that change of place and variety is eminently injurious as a means of cure, until the melancholy hallucinations are completely at an end.

"This is in great contrast with the good which does arise from such a plan in the deep and real affliction which results from loss of friends, simply severe bodily illness, or any great moral distress. In these, if the unhappy individual can be persuaded to travel, new life comes from the exertion.

"The contemplation or the thought of what great changes occur on the face of the earth,—how many thousands have equally great or worse sorrows,—the very distraction of occupation,—works wonders.

"In the one example the mind constantly rebels against the change of scene; in the other, the mind borne down with affliction, still in right-minded people lends it assistance to recover.

"To the really melancholic and hypochondriac it does most serious harm. I speak decidedly, for I have seen many cases retarded in their cure by the prevalent and popular opinion that variety and change of scene will benefit cases of mental aberration, either in the incipient or confirmed stage." P. 180.

The recovery is always much promoted by encouraging the patient to engage in some regular pleasing occupation. For women, Embroidering is one of the very best.

In most of the cases of which we have just been speaking, the catamenial discharge is irregular or altogether deficient. When the mental malady is cured by the use of opiates, &c. the secretion often returns as one of the results of restored health; but this is not always the case. By far the most efficient remedy for the re-establishment of this important function is, in our author's opinion, the direct application of leeches to the cervix uteri, two or three days before the expected period.

"It appears to me," says Dr. Seymour, "to be very desirable to impress upon the profession the great advantage of this practice; it is equally useful in the hysterical mania of young women, which is met with not unfrequently, and here the application of the remedy is still more difficult, and the careful manner of its application still more necessary. I have seen the most serious and alarming illness disappear under the regular adoption of this remedy, and I have every reason to believe that I have seen consequences of the most distressing nature, namely, the establishment of mental derangement, averted by it." P. 184.

Very true; but then the same result may often be obtained by simpler and less objectionable measures. The very case, quoted by our author as an example of its efficacy, shows this; for the patient had recovered on a former occasion without its adoption. How comes it, too, that no chalybeate was ordered in a case of "languid amenorrhœa?" Under such circumstances, the employment of Electricity—shocks passed through the pelvis—is often of the greatest utility.

In the morose and fretful crabbed Melancholy or Hypochondriasis of old age, which, alas! not unfrequently terminates in suicide, the treatment by sedatives, as already explained, will often be found to be most useful. It may be necessary to have recourse to bleeding or cupping, and the use of saline aperients, if there be any symptoms of cerebral congestion; but it will be almost always useful to give from $\frac{1}{4}$ to $\frac{1}{2}$ of a grain of the morphia every night, at bed-time. There is unquestionably far too much dread of Opium in any form, among medical men generally, in cases where they suspect disordered cerebral circulation. As a matter of course, its exhibition requires caution and watchfulness; but this is no more than is necessary in respect of every potent remedy;—potent either for evil or for good, according to the judgment with which it is employed. The case of the poet Crabbe, as related in his life, suggests some useful reflections:

"My father," (says Mr. Crabbe's son,) "now in his forty-sixth year, was much more stout and healthy than when I first remember him. Soon after that early period he became subject to vertigo, which he thought *indicative of a tendency to apoplexy*, and was occasionally *bled rather profusely*, which only increased the symptoms. When he preached his first sermon at Muston in the year 1789, my mother foreboded, as she afterwards told us, that he would preach very few more; but it was on one of his early journeys into Suffolk, in passing through Ipswich, that he had the most alarming attack. Having left my mother at the Sun, he walked into the town alone, and suddenly staggered in the streets, and fell. He was lifted up by the passengers, and overheard some one say significantly, 'Let the gentleman alone, he will better by and by;' for his fall was attributed to the bottle. He was assisted to his room, and the late Dr. Clubbe was sent for, who after a little examination, saw through the case with great judgment. 'There is nothing the matter with your head,' he observed, 'nor any apoplectic tendency; let the digestive organs bear the whole blame! you must take *opiates*.' From that time his health began to amend rapidly, and his constitution was renovated: a rare effect of opium, for that drug almost always inflicts some partial injury, even when it is necessary,* but to him it was only salutary, and to a constant but slightly increasing dose of it may be attributed his long and generally healthy life." P. 163.

Dr. Seymour, we must not omit to remark, has been led by the results of his experience to the conclusion, that one of the most frequent causes of failure in the cure of uncomplicated—*i. e.* unattended with any organic lesion of the encephalon—Melancholy and Hypochondriasis, is the intimate connection between diseases of the mind and diseases of the lungs, especially Consumption. Esquirol has remarked that "les melancholiques succombent presque toujours à des maladies chroniques, particulierement aux affections de poitrine;" and he states that of 176 patients, who died affected by melancholy, 62 were carried off by phthisis. Of the 20 cases, alluded to above, in which the sedative treatment failed of producing decided benefit, a large proportion, 12, proved fatal from this disease. It becomes, therefore, the duty of the physician to have his attention drawn, every now and then, to the state of the thoracic organs in melancholic patients, whose cases are found to resist every method of treatment that can be devised.

Dr. Seymour promises us one or two more volumes of the same general character as the present. They will doubtless be acceptable to the profession; for all his writings shew him to be the shrewd and experienced physician. But we must call upon him to bestow more labour upon their composition, and aim at giving them something better than a merely ephemeral reputation. We cannot admit the plea of "unceasing professional occu-

---

* "This is the opinion of Mr. Crabbe, notwithstanding his father's recovery, not of Dr. Clubbe; and this, in spite of recovery, is the ordinary state of alarm in the minds of people ignorant of medicine—the same as to colchicum, and to many of our most important means of cure.

"I have known at least twenty cases similar to the one related by Mr. Crabbe."

pation and much anxiety" as an apology for the hasty and superficial character of the present volume. He has only to look at the writings of his eminent friend and late colleague at St. George's Hospital, whose time and thoughts must have been quite as much taken up as his own, and he will at once perceive that the excuse put forward is more convenient than well-founded.

